# Impact of Rurality on Total Joint Arthroplasty Access and Outcomes: A Systematic Review

**DOI:** 10.1016/j.artd.2025.101884

**Published:** 2026-03-30

**Authors:** Sean L. Agrodnia, Catherine M. Call, Andrew D. Lachance, John C. McDonald, Adam J. Rana, Brian J. McGrory

**Affiliations:** a Tufts University School of Medicine, Boston, MA, USA; b MMP Orthopedics & Sports Medicine, Maine Medical Center, Portland, ME, USA; c Department of Orthopedic Surgery, Brown University, Providence, RI, USA; d Department of Orthopedic Surgery, Guthrie Clinic, Sayre, PA, USA; e Department of Orthopedics and Physical Rehabilitation, UMassMemorial Medical Center, Worchester, MA, USA

**Keywords:** Total hip arthroplasty, Total knee arthroplasty, Rural, Outcomes, Access, PROMs, Health-care equity

## Abstract

**Background:**

Total joint arthroplasty (TJA) is one of the most frequent orthopaedic surgeries in the United States; however, disparities in utilization and outcomes based on race, sex, and socioeconomic level have been well documented. The impact of rural geographic location, a characteristic that may impact access to TJA care and postoperative outcomes, remains understudied. This systematic review investigated associations between rural location and several metrics in total hip arthroplasty and total knee arthroplasty to investigate whether disparities are present based on rural geographic location.

**Methods:**

In November 2024, PubMed, Web of Science, Scopus, EMBASE, and Cochrane Review databases were queried. Ten studies investigating TJA utilization and outcomes that included patients in a rural location were included. Study quality was assessed using a modified Newcastle-Ottawa Scale.

**Results:**

Ten articles were included in the final analysis. Differences in complications, readmission rates, and length of stay due to rural location of patient and/or hospital were reported. Results indicate urban TJA patients more frequently discharge to rehab. Patient-reported outcome measures following TJA do not appear different between rural and urban patient groups.

**Conclusions:**

Rural geographic location is associated with different TJA utilization and outcomes and may point to disparities in care. Continued efforts dedicated to eliminating inequalities in TJA care should consider the impact geographic location may have on exacerbating other forms of inequity. The reporting of urban-rural classification of patients in arthroplasty research is important for advancing this area of study. Access to care for all patients should be prioritized.

## Introduction and background

Health-care equity requires universal access to total joint arthroplasty (TJA) and its associated quality-of-life improvements. [[1], [2], [3], [4], [5]] Disparities in TJA utilization and outcomes linked to sex, race, and socioeconomic status have been characterized, with recognition of these disparities essential for addressing them and working to improve accessibility within the field of arthroplasty. [[6], [7], [8], [9], [10]] There are much less data available on the impact of geographic location (in particular, rural location) on TJA utilization and patient outcomes.

“Rural,” as it is defined by the US Office of Management and Budget, comprises “all areas outside a metropolitan area.” [11] Nineteen percent of the US population (59.5 million people) resides within a rural area, despite 72% of the US land area qualifying as rural. [12] Rural patients have been shown to have unique health-care risk factors, including poorer metrics of population health and higher prevalence of behavioral risk factors that can impact TJA. [13,14] Rates of diabetes, [15] obesity, [16] and tobacco use [17], factors associated with increased complications and worse outcomes following TJA, [[18], [19], [20]] are notably higher among rural populations. Additionally, many specialist services—including orthopaedic surgery and arthroplasty—are typically located in centralized locations, urban areas, and tertiary care centers. Yet, the aging population is increasing faster in rural areas. [21] Accessing such care centers may be especially challenging for rural TJA patients, who may be older and need nursing care postoperatively. [22] Rural geographic location has a potentially significant impact on health equity, with decreased access to and awareness of health services, lower income, infeasible travel distance, and lack of availability and accessibility of transport disproportionately impacting rural communities. [21] However, the impact of living in rural settings on patient TJA outcomes remains unclear, and as far as we are aware there has been no review of the evidence. This systematic review aims to evaluate TJA utilization and assess whether clinical and patient-reported outcomes following TJA are poorer for individuals living in rural locations.

## Material and methods

### Eligibility criteria and search strategy

A comprehensive literature review was conducted on November 15, 2024, according to the Preferred Reporting Items for Systematic Reviews and Meta-Analyses guidelines [23] using PubMed, EMBASE, Scopus, Web of Science, and Cochrane Review databases. English-language articles examining TJA utilization and outcomes relative to rural geographic location were considered eligible if they met the following criteria: [1] the study employed a prospective or retrospective cohort, case control, or case series design; [2] patients were aged 18 years or older and undergoing primary THA or TKA within the United States; and [3] the authors investigated associations between rural geographic location—of either patient or hospital—and surgical utilization, complications, mortality, length of stay (LOS), discharge disposition, readmission rate, reoperation rate, patient-reported outcome measures (PROMs) or other metric identified in text as a postoperative outcome. Studies including only patients undergoing revision TJA and studies reporting patients outside of the United States were excluded. Conference articles and published abstracts were excluded. Because our focus was on clinical research, not basic science, filters for research on human subjects were employed when available, including ‘human’ filters in PubMed and EMBASE. A human filter was unavailable in Scopus, so search results for the subject area were limited to “medicine” or “health professions.” The comprehensive search strategy employed was comprised of relevant keywords and Medical Subject Headings terms (Appendix A). Included article reference lists underwent subsequent manual inspection to identify any articles not captured by the original computerized search. Articles deemed eligible were published between January 1, 1990, and November 15, 2024.

### Study selection

**Figure 1:**
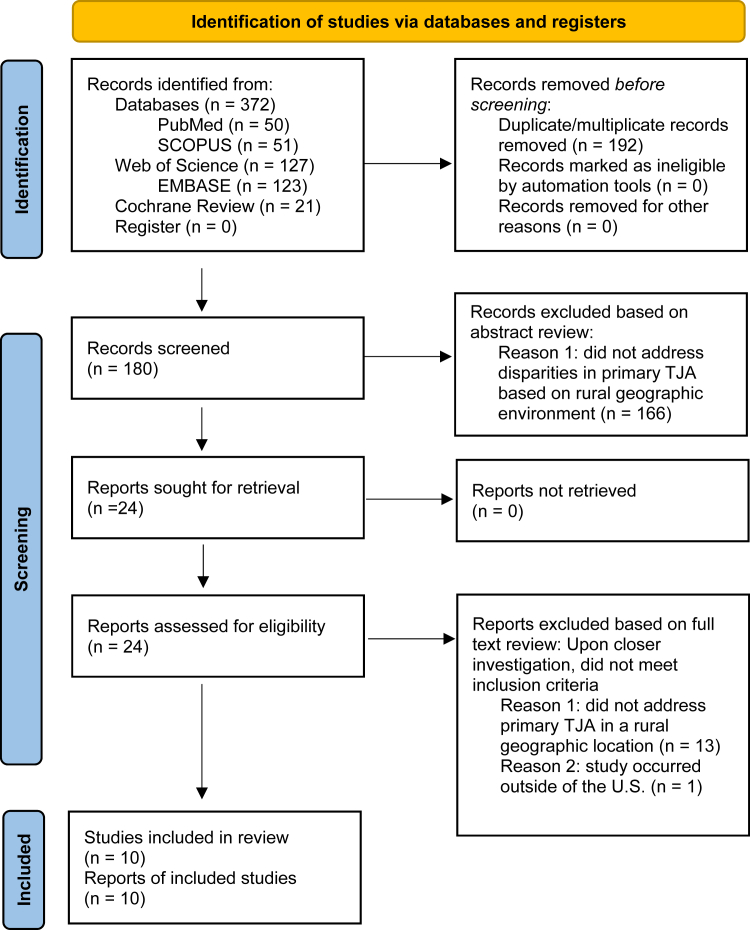
Study flowchart according to Preferred Reporting Items for Systematic Reviews and Meta-Analyses guidelines [22].

This search yielded 372 publications, of which 190 were unique and subsequently screened for inclusion (Fig. 1). Duplicates were identified by 1 author (C.M.C.) using Rayyan (Rayyan Systems, Cambridge, MA). [24] Titles and abstracts of the resulting articles were then independently investigated by 2 authors (S.L.A. and C.M.C.) in Rayyan [24] based on title and abstract. Potentially eligible articles underwent a full-text review by 1 for 4 authors (S.L.A., C.M.C., A.D.L., and J.C.M.) prior to final determination of study inclusion. Reviewers noted any discrepancies and discussed findings to settle disagreements regarding inclusion. Data were recorded, and analysis occurred within Microsoft Excel (Microsoft Inc., Seattle, Washington).

### Assessment of quality

The quality of evidence of included studies was evaluated using a modified Newcastle-Ottawa Scale (NOS) informed by the modification suggested by Rusidill et al, [25] evaluated by 1 author (C.M.C.) (Table 1). The modified NOS [25,26] is a 9-item assessment of internal and external validity based on subject selection, comparability between study groups, and assessment of outcomes, with a maximum possible score of 12. Studies with a score of greater than 10 are considered high quality, while studies scoring between 7 and 9 are considered intermediate quality, and those with a score of less than 7 are of low or questionable quality. [25]

**Table 1:** Study quality appraisal using a modified NOS [24,25].

Criteria	Question
Selection	Was the cohort of patients undergoing total hip or knee arthroplasty representative? (somewhat representative = 1, truly representative = 2)
	Was the selection of the patient cohort drawn from the same community? (yes = 1)
	Was patient information adequately verified? (secure record = 2, structured interview or self-report = 1)
	Did the study demonstrate that the outcome(s) of interest was not present at the start of the study? (yes = 1)
Comparability	Did the study control for age and gender? (yes = 1)
	Did the study control for the presence of comorbid conditions? (yes = 1)
Outcome	Was the outcome(s) of interest clearly defined? (yes = 1)
	Was study follow-up of adequate duration to allow for outcomes to occur? (>30 d = 1)
	Was follow-up adequate? (100% of patients completed follow-up = 2, >90% of patients completed follow-up = 1)

### Analysis

Data were recorded, and analysis occurred within Microsoft Excel (Microsoft Inc, Seattle, WA). Metanalysis was not attempted due to the high heterogeneity across the pooled study data.

## Results

### Demographics

Hospital location vs patient home address-defined rurality is reported alongside the method by which authors determined rurality within their respective studies (Table 2). Journal, study design, level of evidence, modified NOS, procedure type, number of procedures and geographic classification were reported (Table 3). While generally more female patients were undergoing arthroplasty, [[27], [28], [29], [30], [31]] several studies reported rural communities tended to have a higher percentage of males undergoing TJA compared with urban communities. Hinman et al showed 47.2% of rural patients and 40.3% of urban patients were male. [31] Garlapaty et al showed that 38.1% of rural patients and 34.7% of urban patients were male (*P* < .001). [27] Francis et al showed that 43.6% of the patients in the most urban population and 47.4% of the most rural population were male. [32]

**Table 2:** Definition and determination of rurality by individual studies

Article	Rurality defined by patient or hospital location	Determination of rurality utilized
Hinman et al (2023)	Patient	Census derived classification mapped with Zip code tabulation areas
Lachance et al (2023)	Patient	Rural–urban code reported from the US Department of Agriculture Electronic Research Service based on population and proximity to metropolitan area
Ferucci et al (2024)	Patient	Reported from the Inpatient Database of the State of Alaska Health Facilities Data Reporting Program region of residence classification
Hadlandsmyth et al (2024)	Patient	Census driven rurality based on geocoded patient addresses from The Veterans Health AdministrationPlanning Systems Support Group data set
Garlapaty et al (2023)	Patient	Census driven Rural–Urban Commuting Area classifications based on patient zip code
Broggi et al (2022)	Hospital	United States Census Bureau criteria based on hospital zip code
Francis et al (2011)	Patient	Census driven Rural–Urban Commuting Area classifications based on patient zip code
Keeney et al (2017)	Hospital	Classified as high-volume rural tertiary academic hospital with data from the Orthopaedic Operational Data Repository
Freburger et al (2011)	Patient	Patient zip code classified using the National Center for Health Statistics urban/rural classification scheme
Losina et al (2004)	Patient	Patient zip code classified based on Census categorization and population size
Kamath et al (2015)	Patient	Census driven Rural–Urban Commuting Area classifications based on patient address

**Table 3:** Summary of included studies evaluating impact of rural geographic location on TJA utilization and outcomes.

Article	Journal	Study design	Level of evidence	Modified NOS	Procedure	Geographic region assessed	Number of patients	Reported age (y)	Reported sex
Hinman et al (2023)	The Journal of Arthroplasty	Retrospective cohort	4	10	TKA	Urban, Rural, Middle	275, 967	(Median, IQR); Rural (65, 58-72); Middle (65, 59-72); Urban (64, 57-71)	(M%, F%): Rural (47.2, 52.8); Middle (46.2, 53.8); Urban (40.3, 59.7)
			4	10	THA	Urban, Rural, Middle	93,642	(Median, IQR); Rural (67, 60-74); Middle (67, 60-74); Urban (68, 60-75)	(M%, F%): Rural (43.4, 56.6); Middle (44.4, 55.6); Urban (41.0, 59.0)
Lachance et al (2023)	Arthroplasty Today	Retrospective cohort	4	9	THA	Urban, Rural	6066	Mean ± SD: Rural (66 ± 10), Urban (65 ± 11); *P* < .001	(M%, F%): Rural (45,55), Urban (46,44); *P* = .2
Ferucci et al (2024)	Arthritis Care and Research	Retrospective cohort	4	10	TKA	Rural	4594	Alaska Native/American Indian: 1.2% (18-39), 59.2% (40-64), 39.6% (≥65); White: .8% (18-39), 44.9% (40-64), 54.4% (≥65); *P* < .01	(M%, F%): Alaska Native/American Indian (42.1, 57.9), White (45.7, 54.3); *P* < .01
			4	10	THA	Rural	2, 791	Alaska Native/American Indian: 5.2% (18-39), 59.5 (40-64), 35.3 (≥65); White: 2.7 (18-39), 42.8 (40-64), 54.6 (≥65); *P* < .01	(M%, F%): Alaska Native/American Indian (51.6, 48.4), White (48.4, 51.6); *P* < .01
Hadlandsmyth et al (2024)	Journal of Knee Surgery	Retrospective cohort	4	9	TKA	Urban, rural, highly rural	9064	70.59 ± 4.58	(M%, F%) 96.99, 3.01
Garlapaty et al (2023)	Journal of Arthroplasty	Prospective cohort	4	11	TKA	Urban, Suburban, Rural	294	Urban: 63 (35-87); Suburban: 63 (28-85); Rural: 64 (46-89); *P* = .18	(M%, F%): Urban (34.7, 65.3); Suburban (36.8. 63.3); Rural (38.1, 61.9); *P* < .001
Broggi et al (2022)	Journal of Arthroplasty	Retrospective cohort	4	10	THA	Urban, Rural	18,712	Mean ± SD: Urban (65.6 ± 4.9), Rural (65.6 ± 4.9); *P* = .999	(M%, F%): Urban (48.3,51.7), Rural (48.3,51.7); *P* = .999
Francis et al (2011)	Archives of Surgery	Cross-sectional study	4	9	TKA	Urban, Rural	257, 892	Mean (SD) RUCA 1: 71.1 (12.2); RUCA 2: 69.9 (11.8); RUCA 3: 69.7 (12.0); RUCA 4: 70.5 (12.5); RUCA 5: 69.3 (12.2); RUCA 6: 69.3 (12.3); RUCA 7: 70.5 (12.5); RUCA 8 69.1 (12.6); RUCA 9: 69.3 (12.4); RUCA 10: 70.3 (12.1)	(M%, F%) RUCA 1 (43.6, 56.4); RUCA 2: (46.7, 53.3); RUCA 3: (47.1, 52.9); RUCA 4:(44.1, 55.9); RUCA 5: (47.5, 52.5); RUCA 6: (47.4, 52.6); RUCA 7: (44.8, 55.2); RUCA 8 (47.4, 52.6); RUCA 9: (47.5, 52.5); RUCA 10: (47.4, 52.6)
			4	9	THA	Urban, Rural	109, 616		
Keeney et al (2017)	The Journal of Arthroplasty	Prospective cohort	4	10	TKA	Rural	2060 patients with 2198 surgeries	15% (55-59); 18% (60-64); 21% (65-69); 15% (70-74); 10% (75-79); 7% (80+)	(M%, F%) 43, 57
Freburger et al (2011)	Arthritis Care and Research	Cross-sectional study	4	9	THA, TKA	Large, Medium Metropolitan	164, 785	68.1 ± 10.2	(M%, F%) 39.7, 60.3
Losina et al (2004)	Arthritis & Rheumatology	Retrospective cohort	4	11	THA	Urban, Suburban, Rural	1, 149	38% (≥75), 62% (<75)	(M%, F%) 37, 63
Kamath et al (2015)	The Journal of Arthroplasty	Retrospective cohort	4	11	TKA	Urban (nonteaching), Urban (teaching), Rural	24, 422		
					THA	Urban (nonteaching), Urban (teaching), Rural	22, 405		

IQR, interquartile range; M, male; F, female; SD, standard deviation; RUCA, Rural-Urban Commuting Area.

Hinman et al (median interquartile range: rural 65 years vs urban 64 years) [31] and Lachance et al (rural 66 years vs urban 65 years, *P* < .001) [22] showed that urban patients undergoing TJA tended to be younger than rural patients. However, Garlapaty et al (urban 63 years vs rural 64 years, *P* = .18) [27] and Broggi et al (urban 65.6 years vs rural 65.6 years, *P* = .999) [33] showed there was no significant difference in age at time of TJA and a patient’s rurality. Other studies that reported age did not directly compare age of rural and urban patients.

### Utilization

Hinman et al found that TKA utilization was lower in urban compared to rural populations, with an adjusted incidence rate ratio of 0.88 (95% confidence interval: 0.82-0.95) for urban vs rural settings. [22] THA utilization differences were similarly pronounced, with urban settings demonstrating an incidence rate ratio of 0.87 (95% confidence interval: 0.81-0.94). [22] Losina et al found difference in utilization of low-volume hospitals (risk ratio, *P* value) depending upon patient residency; rural vs urban (3.50, <0.0001), rural vs suburban (2.00, <0.0001), and suburban vs urban (1.74, <0.0001). [30] Francis et al found higher utilization in rural patients for both TKA (rural vs urban, 1.15 (1.13-1.16)) and THA (rural vs urban, 1.15 (1.13-1.18)). [32]

### Outcome measures

Few differences were found in complication rates among urban and rural patient cohorts in several studies (Table 4, Table 5). Lachance et al found complications of rural patients to be 1.2% vs 1.4% within the urban population (*P* = .4). [22] Broggi et al found higher rates of complications (urban%, rural%, *P* value) within the rural group in terms of dislocation (1.11, 1.39, *P* < .001), revision surgery (1.09, 1.29, *P* = .019), prosthetic joint infection (0.84, 0.96, *P* = .023), return to OR for irrigation and debridement (1.03, 1.14, *P* = .035), and wound complications (0.98, 1.05, *P* = .044). [33] There was no difference in terms of sepsis (0.54, 0.58, *P* = .717), myocardial infarction (0.59, 0.68, *P* = .379), stroke (1.53, 1.47, *P* = .713), thromboembolic event (1.55, 1.62, *P* = .594), and pneumonia (0.52, 0.48, *P* = .556) between the urban and rural groups. [33]

**Table 4:** Summary of the outcomes evaluated by included TJA studies.

Article	Outcome measured							
	Utilization	Complications	Mortality	Length of stay	Discharge disposition	Readmission	Reoperation	PROMs
Broggi et al (2022)	**-**	**+**	**+**	**+**	**+**	**+**	**-**	**-**
Ferucci et al (2024)	**+**	**-**	**-**	**+**	**+**	**-**	**-**	**-**
Francis et al (2011)	**+**	**-**	**-**	**-**	**-**	**-**	**-**	**-**
Freburger et al (2011)	**-**	**-**	**-**	**-**	**+**	**-**	**-**	**-**
Garlapaty et al (2023)	**-**	**-**	**-**	**-**	**-**	**-**	**-**	**+**
Hadlandsmyth et al (2024)	**-**	**+**	**-**	**-**	**-**	**-**	**-**	**-**
Hinman et al (2023)	**+**	**-**	**-**	**-**	**-**	**-**	**-**	**-**
Keeney et al (2017)	**-**	**-**	**-**	**+**	**+**	**-**	**-**	**+**
Lachance et al (2023)	**-**	**+**	**-**	**+**	**+**	**+**	**+**	**+**
Losina et al (2004)	**+**	**-**	**-**	**-**	**-**	**-**	**-**	**-**

**Table 5:** Outcome results of included TJA studies.

Article	Utilization	Complications	Length of stay	Discharge disposition	Readmission	Reoperation/Surgical Admission	PROMs
Hinman et al (2023)	TKA (Urban vs Rural) = 0.88 (0.82-0.95) (IRR)	**-**	**-**	**-**	**-**	**-**	**-**
	TKA (Middle vs Rural) = 1.07 (0.95-1.20) (IRR)	**-**	**-**	**-**	**-**	**-**	**-**
	THA (Urban vs Rural) = 0.87 (0.81-0.94) (IRR)	**-**	**-**	**-**	**-**	**-**	**-**
	THA (Middle vs Rural) = 1.01 (0.90-1.14) (IRR)	**-**	**-**	**-**	**-**	**-**	**-**
Lachance et al (2023)	**-**	Rural = 1.2%	Rural = 34 h	Rural = 59% (Home or self-care, 34% (Home health care), 5.6% (Skilled nursing facility), 1.1% (Rehab facility)	Rural (90 d) = 2.0%	-	**+**
		Urban = 1.4%	Urban = 35 h	Rural = 60% (Home or self-care, 31% (Home health care), 6.8% (Skilled nursing facility), 1.4% (Rehab facility)	Urban (90 d) = 4.1%	-	+
		*P* = .4	*P* = .002	*P* = .035	*P* < .001		
Ferucci et al (2024)	TKA Alaska Native/American Indian = 0.130%	**-**	TKA Alaska Native/American Indian = 67.0% ≤ 3 d	TKA Alaska Native/American Indian = 97.8% Discharge to home	**-**	**-**	**-**
	THA Alaska Native/American Indian = 0.080%		THA Alaska Native/American Indian = 67.3% ≤ 3 d	THA Alaska Native/American Indian = 96.1% Discharge to home	**-**	**-**	**-**
	TKA White = 0.234%	**-**	TKA White = 81.7% ≤ 3 d	TKA Alaska Native/American Indian = 94.6% Discharge to home	**-**	**-**	**-**
	THA White = 0.147%		THA White = 83.3% ≤ 3 d	THA White = 95.3% Discharge to home	**-**	**-**	**-**
	-	**-**	TKA and THA *P*= <0.01	TKA and THA *P*= <0.01	**-**	**-**	**-**
Hadlandsmyth et al (2024)	**-**	OR (95% CI) Rural, model controlling for social determinants of health: 0.78 (0.62, 0.98) risk of developing long-term opioid use	**-**	**-**	**-**	**-**	**-**
Garlapaty et al (2023)	**-**	**-**	**-**	**-**	**-**	**-**	Rural Preop: 47.2 ± 11.2 (KOOS JR), 40.0 ± 6.7 (PROMIS Global Physical Health), 4.0 ± 1.7 (UCLA Activity Scores), 6.1 ± 2.6 (VAS), 47.9 ± 8.5 (PROMIS Global Mental Health)
	**-**	**-**	**-**	**-**	**-**	**-**	Rural 1 year: 74.1 ± 17.1 (KOOS JR), 47.3 ± 8.0 (PROMIS Global Physical Health), 4.8 ± 1.7 (UCLA Activity Scores), 2.6 ± 2.6 (VAS), 51.1 ± 8.5 (PROMIS Global Mental Health)
	**-**	**-**	**-**	**-**	**-**	**-**	Suburban Preop: 44.9 ± 13.7 (KOOS JR), 39.0 ± 6.4 (PROMIS Global Physical Health), 4.3 ± 3.5 (UCLA Activity Scores), 5.9 ± 2.7 (VAS), 48.2 ± 8.1 (PROMIS Global Mental Health)
	**-**	**-**	**-**	**-**	**-**	**-**	Suburban 1 year:72.4 ± 19.6 (KOOS JR), 45.7 ± 8.6 (PROMIS Global Physical Health), 4.9 ± 1.8 (UCLA Activity Scores), 2.9 ± 2.9 (VAS), 49.7 ± 8.4 (PROMIS Global Mental Health)
	**-**	**-**	**-**	**-**	**-**	**-**	Urban Preop: 48.9 ± 14.0 (KOOS JR), 40.0 ± 7.0 (PROMIS Global Physical Health), 4.3 ± 4.2 (UCLA Activity Scores), 5.9 ± 2.7 (VAS), 48.5 ± 9.5 (PROMIS Global Mental Health)
	**-**	**-**	**-**	**-**	**-**	**-**	Urban 1 year: 73.3 ± 17.1 (KOOS JR), 46.4 ± 8.7 (PROMIS Global Physical Health), 5.0 ± 1.7, UCLA Activity Scores, 2.8 ± 2.8 (VAS), 50.3 ± 9.2 (PROMIS Global Health)
Broggi et al (2022)	**-**	Surgical Complications (Urban%, Rural%, *P*-value): Dislocation (1.11, 1.39, <.001); Revision Surgery (1.09, 1.29, .019); Prosthetic Joint Infection (0.84, 0.96, .023); Return to OR for I&D (1.03, 1.14, .035); Wound Complications (0.98, 1.05, .044)	Extended LOS ≥3 d (Urban%, Rural%, *P*-value): 9.37, 13.89, <0.001	Nonhome Discharge (Urban%, Rural%, *P*-value): 8.28, 8.21, .573	30 d Readmission (Urban%, Rural%, *P*-value): 3.11, 4.28, 0.031	**-**	**-**
	-	Medical Complications (Urban%, Rural%, *P*-value): Sepsis (0.54, 0.58, 0.717); Myocardial Infarction (0.59, 0.68, 0.379); Stroke (1.53, 1.47, 0.713); Thromboembolic Event (1.55, 1.62, 0.594); Pneumonia (0.52, 0.48, 0.556)	**-**	**-**	90 d Readmission (Urban%, Rural%, *P*-value): 5.03, 6.58, 0.018	**-**	**-**
Francis et al (2011)	TKA (Rural vs Urban) = 1.15 (1.13-1.16) (OR)	**-**	**-**	**-**	**-**	**-**	**-**
	THA (Rural vs Urban) = 1.15 (1.13-1.18) (OR)	**-**	**-**	**-**	**-**	**-**	**-**
Keeney et al (2017)	**-**	**-**	LOS ≥3 d (OR, *P*-value): Minority identity (2.39, 0.003); Income <35k (2.74, 0.017)	Facility/Rehab Discharge (OR, *P*-value): Minorities (4.43, *P* < .001); Live Alone (2.62, *P* < .001); Unemployed (1.78, 0.031)	**-**	**-**	Negative PROMs (OR, *P*-value): Not Working (0.55, 0.031)
Freburger et al (2011)	**-**	**-**	**-**	Institution vs Home Discharge (OR, *P*-value): Micropolitan/Rural (1.00, -), Medium (1.06, .0879), Large (1.27, <0.0001)	**-**	**-**	**-**
	**-**	**-**	**-**	HH vs Home Discharge (OR, *P*-value): Micropolitan/Rural (1.00, -), Medium (1.27, <0.0001), Large (1.40, <0.0001)	**-**	**-**	**-**
	**-**	**-**	**-**	SNF vs IRF (OR, *P* value): Micropolitan/Rural (1.00, -), Medium (1.25, 0.005), Large (1.52, <0.0001)	**-**	**-**	**-**
Losina et al (2004)	Low-volume hospital use (RR, *P* value): Rural vs Urban (3.50, <0.0001); Rural vs Suburban (2.00, <0.0001); Suburban vs Urban (1.74, <0.0001)	**-**	**-**	**-**	**-**	**-**	**-**

IRR, incident rate ratio; OR, odds ratio; CI, confidence interval; I&D, irrigation and debridement; SNF, skilled nursing facility; IRF, independent rehabilitation facility; HH, home health; RR, relative rate.

Lachance et al found urban patients stayed slightly longer postoperatively (35 hours in the urban group vs 34 hours in the rural group, *P* = .002) with more urban patients going to skilled nursing facilities (6.8% vs 5.6%, *P* = .035). [22] Extended LOS ≥3 days was more frequent in rural population in the study by Broggi et al (urban 9.37%, rural 13.89%, *P* < .001). [33] Within a rural population, LOS ≥3 days was higher for patients with a minority identity (2.39, *P* = .003) and patients with an income less than 35,000 dollars (2.74, *P* = .017). [27] Ferucci et al also found that patients who identified as White were more likely to be discharged with a LOS ≥3 days for TKAs (81.7% vs 67.0%, *P* < .01) and THAs (83.3%, 67.3%, *P* < .01) in comparison to Alaska Native or American Indian–identifying patients who were more associated with living in rural areas. [34] Other studies found no difference in nonhome discharge in urban vs rural patients (8.28%, 8.21%, *P* = .573). [33] Within the rural patient population, Keeney et al found facility or rehabilitation discharge to be higher for patients with minority identities (4.43, *P* < .001), patients who lived alone (2.62, *P* < .001); and those who are unemployed (1.78, *P* = .031). [28] Freburger et al similarly found that urban patients more likely to be discharged to an institution vs home, ((Micropolitan/Rural (1.00, -), Medium (1.06, 0.0879), Large (1.27, <0.0001)), have home health vs home discharge (Micropolitan/Rural (1.00, -), Medium (1.27, <0.0001), Large (1.40, *P* < .0001)), and go to a skilled nursing facility vs independent rehabilitation facility (OR, *P* value): Micropolitan/Rural (1.00, -), Medium (1.25, 0.005), Large (1.52, *P* < .0001)). [29] Similarly, Ferucci et al found that patients who identified as Alaska Native or American Indian were more likely to live in rural areas and more frequently were discharged to home when compared to White-identifying patients for TKA (97.8%, 94.6%, *P* < .01) and THA (96.1%, 95.3%, *P* < .01). [34]

Lachance et al reported that readmission at 90 days was higher in the urban population at 4.1% vs 2.0% in the rural population (*P* < .001). [22] This is in contrast to Broggi et al, which found higher readmission rates in rural populations at 30 and 90 days (3.11, 4.28, *P* = .031) and (5.03, 6.58, *P* = .018). [33]

### PROMS

Garlapaty et al reported no difference in Knee injury and Osteoarthritis Outcome Score Joint Replacement (KOOS JR) scores at baseline (*P* = .20) or at 1 year (*P* = .66). [27] Similarly, Patient-Reported Outcomes Measurement Information System (PROMIS) Global Physical Health, University of California, Los Angeles Activity Score, changes in Visual Analog Scale (VAS), and PROMIS Global Mental Health scores were not significantly different between groups preoperatively or at 1 year. [27] Lachance et al found no difference in Hip Disability and Osteoarthritis Outcome Score, Joint Replacement (*P* = .47), single assessment numeric evaluation (*P* = .84) and PROMIS (*P* = .31) scores at 1 year between rural and urban patients (Table 5). [22]

## Discussion

We examined the differences in utilization, hospital, and patient-reported outcomes in rural and urban populations undergoing THA and TKA within the United States to evaluate for differences that may be attributable to geographic location and availability of resources and care. Arthroplasty utilization has generally been demonstrated to be higher within rural populations. [31,34] Yet, differences in complications due to rural location of patient and/or hospital is unclear. Based on our analysis of available studies addressing this question, urban patients were more likely to go to inpatient rehabilitation facilities. [29,35] We identified no clear trends regarding readmission rates and LOS, and there were no clearly identifiable differences in PROMs between rural and urban patient groups.

### Utilization

Utilization of TJA was generally higher within rural populations, with 3 studies reporting rural patients were more likely to undergo TJA. [31,32,36] This is substantiated by previous literature demonstrating rural patients have higher rates of utilizing arthroplasty. [34,37] These findings are likely multifactorial. Rural patients have been found to be slightly older, more likely to be on government insurance, and have a higher need for nursing assistance postoperatively. [22] Older patients requiring more nursing assistance suggests these patients are presenting late into the disease process and may be more likely to require surgical intervention. Rural patients with lower income may struggle to access medical care due to difficulty accessing transportation and other resources required to travel to health-care setting, which may impact overall health status and the progression of arthritis by time of presentation to an arthroplasty surgeon as well. [38,39]

### Outcome measures

Rates and incidence of complications among urban and rural arthroplasty patients differed between several studies. Lachance et al [22] found similar complication rates between urban and rural patients undergoing THA. Alternatively, Broggi et al [33] found higher rates of dislocation, revision, prosthetic joint infection, return to OR, and wound complications occurring within the rural group following primary THA. A previous study found that rural hospitals had a higher incidence of periprosthetic fracture following primary THA, which was not replicated in any studies within our review. [40] It is unclear if complications are more related to risk factors of rural patient populations or rural hospitals themselves. Rural hospitals, which are often also designated as critical access hospitals, often have lower volume and fewer resources, which may impact outcomes and variation in performance when compared to high-volume hospitals and centers that may be more familiar with caring for TJA patients. [[41], [42], [43], [44], [45], [46]] The role of rurality relating to complications is still unclear following THA and TKA. Studying outcomes in this patient population is complicated by difficulty is classifying patients by both rural home location and location of care; rural patients receiving care locally may be treated in lower volume hospitals who less frequently care for TJA patients, while those receiving care at major centers may incur risks associated with travel and face logistical challenges making preoperative and postoperative office follow-up.

Reports on LOS and discharge disposition for urban and rural TJA patients reveal mixed results. Lachance et al [22] found a slightly longer LOS in urban patients. Nevertheless, Broggi et al [33] found rural patients more likely to stay beyond 3 days, with Keeney et al [28] demonstrating that LOS >3 days following primary TKA was more likely to occur in rural patients with minority racial identity and/or low-income status. Ferucci et al also found a higher percentage of minority identifying patients, who were also associated with living in more rural areas, requiring a LOS greater than 3 days. [34] These results align with a previous study that identifies lower income and minority racial identity as risk factors for increased LOS following TKA. [13] Transportation and access to care may be barriers for rural patients to receive care at tertiary care centers to undergo arthroplasty. Differences in socioeconomic factors including income and availability of family support may also have a larger role in LOS than patient rurality.

In terms of disposition, discharge to rehab was higher among the urban patient population in 2 studies [22,29,47] and not significantly different between groups in 1 study. [33] This decisive finding may be impacted by a variety of factors, including patient selection and facility availability. TJA patients from rural areas that are sicker and with more comorbidities [13,14]—who would be candidates for rehab following TJA—may struggle to access orthopaedic and arthroplasty evaluation, and as such, are not represented in these data because they do not go on to receive TJA due to extensive barriers in accessing TJA services. Rural patients that can access TJA and travel to tertiary care centers where care is provided may be inherently healthier, and thus not require rehab services and need less support postoperatively. Rural patients in the study by Lachance et al [22] were significantly younger when they underwent TJA, which may correlate to a higher overall health status and greater likelihood of discharge home. [48] Secondly, the limited availability of rehabilitation services in rural regions compared to urban areas likely impacts this finding.

### PROMs

Generally, no differences in PROMs were observed between rural and urban populations. Lachance et al [22] found that THA patients from rural areas had higher PROMs scores—including Hip Disability and Osteoarthritis Outcome Score, Joint Replacement and pain, satisfaction, functional improvement, and surgery meeting expectations—at 6 months. However, these values likely did not meet minimal clinically important differences [49] and were largely equivalent with PROMs from the urban group by 1 year. Garlapaty et al [27] found similar KOOS JR, VAS, and PROMIS scores between urban and rural TKA patients 1 year. These results align with a large study performed in Australia, which found no difference in pain and function scales between urban and rural patients at 12 and 24 months following TJA at a large, tertiary academic center. [50] Together, such findings support the goal that patients receiving arthroplasty care at the same hospital—regardless of their home’s geographic zip code and distance traveled to receive care—experience similar PROMs and satisfaction. This is contradictory to a Medicare study, which found that increased rurality lead to decreased satisfaction with health-care services, mainly due to travel distance. [51] However, these findings may be altered by the perceptions of patients receiving the care; if patients believe the quality of the arthroplasty care they are receiving at a larger tertiary care center further away from home is higher, they will likely have better patient-reported outcomes. [52] Multidisciplinary and high volume care has been shown to improve patient satisfaction across hospital care [53,54] and within arthroplasty specifically. [[55], [56], [57]] Thus, rurality does not appear to negatively affect patient-reported outcomes.

### The future and telehealth

One proposed intervention for addressing geographic disparities in arthroplasty—and more widely, medical—care, borne out of the COVID-19 pandemic, is the rise of telehealth services. Telehealth has been shown to enhance access to care for patients in rural areas by reducing travel time and associated costs, which are significant barriers to accessing in-person care. [58] For instance, Butzner and Cuffee highlighted that telehealth interventions across a range of therapeutic areas implemented in rural communities led to increased satisfaction and improved access to care. [58] Additionally, Wongworawat et al noted that telehealth offers opportunities for improved patient care in orthopaedics, with decreased travel time and travel costs to patients offering the potential to make care more accessible and convenient. [59] Trials have identified the feasibility of such telehealth interventions; Moore et al found no significant differences in PROMs between telemedicine and in-person visits following TJA. [60] Similarly, Buvik et al reported no differences in PROMs between video-assisted remote consultations and standard face-to-face general orthopaedic consultations. [61] The future of such interventions, and the logistics associated with video-based care, remains an ongoing and promising area of arthroplasty research with the potential to mitigate disparities in health-care access while providing high-quality and high-value care. [62]

### Limitations and the area deprivation index (ADI)

This study is notable for several possible limitations, many of which are inherent the systematic review design. Pertinent articles may have been missed by this investigation and not captured by our search parameters. Additionally, we were unable to capture articles published in journals not indexed within the databases queried. The strength of the conclusions of any systematic review is dependent on the quality of evidence of included studies, which we evaluated with a modified NOS score. Most studies included were of intermediate to high quality, and the average modified NOS score was 9.9 of 12. The small number of studies that met inclusion criteria and the wide range of reported metrics meant the utility of additional analysis was limited. Additionally, due to a lack of standardization in adjusted analyses across the included studies, reported results may be affected by residual confounding not addressed here. Because this review evaluated study results without regard for year of publication, conclusions concerning changes over time cannot be drawn. Due to the unique characteristics of the US health-care system and concerns regarding comparability between countries, only studies from the US were included, limiting generalizability of findings.

One aspect of this study that we feel is a strength is the inclusion of the area deprivation index (ADI) within the terms of our search parameters. The ADI is a composite measure that quantifies socioeconomic disadvantage at a neighborhood level using a geographic model of zip code tabulation areas and 17 census-based markers of material deprivation and poverty. [63,64] It is reported on a scale from 0 to 100, with higher scores indicating greater disadvantage. [63,64] We evaluated articles addressing the ADI within the scope of our search in order to include any that mentioned rurality or a rural zip code, as determined by the 2013 rural–urban code classifications by the US Department of Agriculture Electronic Research Service. [65] The inclusion of the ADI in our search strategy is likely one of the reasons the number of included articles relative to the number of articles screened was low (5.79%), as most articles reporting on the ADI were excluded because they did not address nor report rural status nor rural zip code. Such a finding indicates that while increased utilization of the ADI in arthroplasty research is an important step toward evaluating the global impact of disadvantaged socioeconomic status on TJA utilization and outcomes, this metric does not necessarily capture the impact of rurality, and findings influenced by rurality are likely limited by lack of reporting.

## Conclusions

We have identified through a systematic review of the literature differential TJA utilization and outcomes based on rural geographic location that may point to disparity in care among primary TJA patients. Additional research in this area of study is needed, and continued reporting of geographic location in arthroplasty research is essential to address these questions. The need exists to define and report rurality consistently to allow for comparison across studies to fully explore the complex interplay between rurality and health outcomes in arthroplasty care, as we work as a field to prioritize and ensure care equity.

## CRediT authorship contribution statement

**Sean L. Agrodnia:** Writing – review & editing, Writing – original draft, Visualization, Validation, Methodology, Investigation, Formal analysis, Data curation. **Catherine M. Call:** Writing – review & editing, Writing – original draft, Visualization, Validation, Supervision, Project administration, Methodology, Investigation, Formal analysis, Data curation, Conceptualization. **Andrew D. Lachance:** Writing – review & editing, Writing – original draft, Visualization, Validation, Methodology, Investigation, Formal analysis, Conceptualization. **John C. McDonald:** Writing – review & editing, Writing – original draft, Visualization, Validation, Methodology, Investigation, Formal analysis. **Adam J. Rana:** Writing – review & editing, Writing – original draft, Validation, Supervision, Project administration, Investigation, Conceptualization. **Brian J. McGrory:** Writing – review & editing, Writing – original draft, Validation, Supervision, Project administration, Investigation, Conceptualization.

## Conflicts of interest

Adam J. Rana receives royalties from, is on the speakers' bureau/paid presentations for, and is a paid consultant for Smith & Nephew; receives research support from Zimmer; and is a Board member/committee appointments for the Eastern Orthopedics Association and AAHKS.

Brian J. McGrory receives royalties from and is a paid consultant for Smith & Nephew; is on the Speakers bureau/paid presentations for Smith & Nephew, Inc., Innomed, Inc., and Springer, Inc.; receives Institutional support from Zimmer Biomet; receives royalties, financial or material support from Springer, Inc.; and is on the Medical/Orthopaedic publications editorial/governing board for Arthroplasty Today (AAHKS).

The other authors declare no potential conflicts of interest.

For full disclosure statements refer to https://doi.org/10.1016/j.artd.2025.101884.

## Appendix A Supplementary Data

Supplemental Rural Arthroplasty

Conflict of Interest Statement for Agrodnia

Conflict of Interest Statement for Call

Conflict of Interest Statement for Lachance

Conflict of Interest Statement for McDonald

Conflict of Interest Statement for McGrory

Conflict of Interest Statement for Rana
