# Supplementary material for: Impact of Rurality on Total Joint Arthroplasty Access and Outcomes: A Systematic Review
Source: Arthroplast Today. 2026 Mar 30;37(Suppl):101884. doi: 10.1016/j.artd.2025.101884 (PMC13081216; doi:10.1016/j.artd.2025.101884)
Supplement: Supplemental Rural Arthroplasty [file mmc1.docx]

**Appendix A.** Search Strategy.

**Web of Science:**

(arthroplasties OR arthroplasty OR replacement) AND (hip OR knee) AND (outcome* OR survival OR vital statistics OR life expect* OR life year* OR morbidit* OR mortalit* OR incidenc* OR prevalenc* OR convalescence OR death* OR quality of life OR life qualit* OR reoperat* OR surgical revis* OR surgery repeat* OR repeat surger* OR repeated surger* OR revised surger* OR repeated operation* OR revised operation* OR patient satisfact* OR patient prefer* OR recovery of function* OR function recover* OR functional recover* OR physical function*) AND (rural OR area deprivation index)

**SCOPUS:**

Title, abstract, keywords: (arthroplasty OR arthroplasties OR replacement) AND (hip OR knee) AND outcome AND (rural OR area AND deprivation AND index)

**EMBASE:**

(arthroplasties:ti,ab,kw OR arthroplasty:ti,ab,kw OR replacement:ti,ab,kw) AND (hip:ti,ab,kw OR knee:ti,ab,kw) AND (outcome*:ti,ab,kw OR survival:ti,ab,kw OR 'vital statistics':ti,ab,kw OR 'life expect*':ti,ab,kw OR 'life year*':ti,ab,kw OR morbidit*:ti,ab,kw OR mortalit*:ti,ab,kw OR incidenc*:ti,ab,kw OR prevalenc*:ti,ab,kw OR convalescence:ti,ab,kw OR death*:ti,ab,kw OR 'quality of life':ti,ab,kw OR 'life qualit*':ti,ab,kw OR reoperat*:ti,ab,kw OR 'surgical revis*':ti,ab,kw OR 'surgery repeat*':ti,ab,kw OR 'repeat surger*':ti,ab,kw OR 'repeated surger*':ti,ab,kw OR 'revised surger*':ti,ab,kw OR 'repeated operation*':ti,ab,kw OR 'revised operation*':ti,ab,kw OR 'patient satisfact*':ti,ab,kw OR 'patient prefer*':ti,ab,kw OR 'recovery of function*':ti,ab,kw OR 'function recover*':ti,ab,kw OR 'functional recover*':ti,ab,kw OR 'physical function*':ti,ab,kw) AND (rural:ti,ab,kw OR 'area deprivation index':ti,ab,kw)

**PubMed:**

((((arthroplasties[Abstract] OR arthroplasty[Abstract] OR replacement[Abstract])) AND (hip[Abstract] OR knee[Abstract])) AND outcome[Abstract]) AND (rural[Abstract] OR area deprivation index[Abstract])

**Cochrane Review:**

((((arthroplasties[Abstract] OR arthroplasty[Abstract] OR replacement[Abstract])) AND (hip[Abstract] OR knee[Abstract])) AND outcome[Abstract]) AND (rural[Abstract] OR area deprivation index[Abstract]) in Title Abstract Keyword
